# Decreased expression of microRNA-124 is an independent unfavorable prognostic factor for patients with breast cancer

**DOI:** 10.1186/s13000-015-0257-5

**Published:** 2015-04-29

**Authors:** Liang-liang Dong, Li-ming Chen, Wei-min Wang, Liang-ming Zhang

**Affiliations:** Department of Medical Oncology, Yantai Yuhuangding Hospital, 20 Yuhuangding East Road, Yantai, Shandong 264000 China

**Keywords:** MicroRNA, MicroRNA-124, Breast cancer, Prognosis, Biomarker

## Abstract

**Background:**

MicroRNA-124 (miR-124) has been reported to be downregulated in breast cancer. However, its clinical significance and prognostic value in breast cancer have not been extensively studied.

**Methods:**

The tissue expression levels of miR-124 were measured using quantitative real-time PCR in 133 breast cancer patients. The correlation between the miR-124 levels and the clinicopathological factors of the patients was also analyzed. Survival and Cox proportional-hazards regression analyses were performed to determine the correlation between miR-124 expression levels and prognosis in the patients.

**Results:**

Quantitative real-time PCR analysis showed that miR-124 had lower expression in breast cancer specimens than that in matched adjacent normal breast tissues (0.39 ± 0.16 vs. 1.00 ± 0.39; P < 0.05). Low miR-124 expression level was significantly associated with advanced TNM stage (P = 0.011), lymph node metastasis (P = 0.012), and poorer pathological differentiation (P = 0.023). A significant difference was found that breast cancer patients with low miR-124 expression level had distinctly shorter overall survival than patients with high miR-124 expression level (63.8% vs. 35.2%, P = 0.03). Furthermore, multivariate analysis of the prognosis factors with a Cox proportional hazards model confirmed that low miR-124 expression was a significant independent predictor of poor survival in breast cancer (HR = 3.16, 95% CI: 1.79-9.13, P = 0.017).

**Conclusion:**

These findings proved that the decreased expression of miR-124 might be associated with tumor progression and poor prognosis in patients with breast cancer.

**Virtual Slides:**

The virtual slide(s) for this article can be found here: http://www.diagnosticpathology.diagnomx.eu/vs/3752603721493544

## Background

Breast cancer ranks as the most common cancer and the first major cause of cancer death among women in the world, with an expected 1,383,500 newly diagnosed cases and 458,400 deaths in 2010 [1]. It is necessary to further explore the molecular mechanisms of breast cancer in order to improve the therapeutic effect. MicroRNAs (miRNAs) are short (~19–23 nucleotides), non-coding RNA molecules that are recognized as endogenous physiological regulators of gene expression. These small RNAs are capable of controlling gene expression either by repressing translation and transcription [2], or by activating transcription [3]. Dysregulation of miRNA expression has been found in various types of human cancers, including breast cancer, colon cancer, and lung cancer, chronic lymphocytic leukemia and malignant glioma [4-8]. MiRNA expression profiles that are able to further distinguish between normal breast tissue and breast cancer have been presented by several groups [7,9]. These differences in expression for certain miRNAs in breast cancer suggest that miRNAs can be useful biomarkers for breast cancer progression and clinical course or may even be key molecular events in the transformation process.

MicroRNA-124 (miR-124), a brain-enriched miRNA, was first found to be involved in stem cell regulation and neuro-development [10,11]. Previous research confirmed that miR-124 was epigenetically silenced in various types of cancers [12-16]. The study by Li et al showed that miR-124 was downregulated in breast cancer, and miR-124 might be a tumor suppressor in breast cancer via the regulation of flotillin-1(FLOT1) [17]. In the present study, we aimed to investigate the clinical significance and prognostic value of miR-124 in breast cancer.

## Methods

### Ethics statement, patients and tissue specimens

The use of tissues for this study has been approved by the Ethics Committee of Yantai Yuhuangding Hospital. At the time of initial diagnosis, all patients had provided consent in the sense that their tumor samples could be used for investigational purposes. Written informed consents were received from all participants involved in the study. Breast cancer tissues and adjacent normal tissues were obtained from 133 patients. All patients underwent breast surgical resection at the Surgery Department, Yantai Yuhuangding Hospital from April 2006 to March 2012. The carcinomas and the adjacent normal tissues were snap-frozen in liquid nitrogen (N2) and stored at -80°C until use. All the specimens were diagnosed by two pathologists separately to determine the pathological classification of breast cancer. None of the patients recruited in this study had undergone preoperative chemotherapy or radiotherapy. The clinical features of the patients, including age, tumor size, histologic grade, lymph node status, and histology, were shown in Table 1.

**Table 1:** **Correlation between miR-124 expression and characteristics of breast cancer patients**

**Clinicopathological factors**	**Cases number**	**miR-124 expression level**		**P value**
		**Low (n = 66)**	**High (n = 67)**	
Age				
<50 years	56	21	35	0.19
≥50 years	77	45	32	
Tumor size				
<2.5 cm	70	27	43	0.13
≥2.5 cm	63	39	24	
Lymph node metastasis				
Yes	59	41	18	0.012*
No	74	25	49	
TNM stage				
I/II	92	35	57	0.011*
III	41	31	10	
Pathological differentiation				
Moderately and highly differentiated	100	45	55	0.023*
Poorly differentiated	33	21	12	
ER				
Positive	67	27	40	0.31
Negative	66	39	27	
PR				
Positive	59	25	34	0.15
Negative	74	41	33	
HER2 status				
Over expressed	65	29	36	0.08
Negative	68	37	31	

* P <0.05 was considered significant.

### Reverse transcription and quantitative real-time PCR

Total RNA and enrichment of small RNA from fresh samples was isolated using the mirVana miRNA Isolation kit (Ambion, Austin, TX, USA) according to the manufacturer’s instructions, and then stored at -70°C until use. Total RNA from fresh cultured cells was carried out with TRIzol reagent (Invitrogen, Karlsruhe, Germany) following the manufacturer’s protocol. Real-time RT-PCR method was used to assess the expression levels of miR-124 with Express SYBR® GreenER qPCRs supermix Universal kit (Invitrogen) on a Rotor-gene 6000 system (Qiagen, Valencia, CA, USA). U6 RNA was used as an endogenous reference for normalizing the expression levels of miR-124. Initially, we calculated a ^Δ^Ct (target-reference), which is equal to the difference between threshold cycles for miR-124 (target) and those for U6 RNA (reference). The fold-change between cancer tissues and normal breast tissue control for miR-124 was calculated with the 2^ΔΔ^Ct method, in which ^ΔΔ^Ct = ^Δ^Ct (target-reference in tumor samples) - ^Δ^Ct (target-reference in normal samples). The relative expression levels of miRNAs in cancer compared to their non-tumorous controls were calculated using the method of 2^-ΔΔ^Ct . The quantitative real-time PCR primers for miR-124 were designed as follows: forward: 5′‑GATACTCATAAGGCACGCGG‑3′ and reverse: 5′‑GTGCAGGGTCCGAGGT‑3′.

### Statistical analysis

All statistical analyses were performed using the SPSS 18.0 statistical software package (SPSS Inc., Chicago, IL, USA). The Student’s t-test was used to analyze the differences in miR-124 expression between the tumor and normal tissues. Relationships between miR-124 expression level and the clinicopathological characteristics were studied using the chi-square test and Fisher’s exact test or independent t test. Survival curves were plotted by the Kaplan-Meier method and compared by the log-rank test. The survival data were evaluated by univariate and multivariate Cox regression analyses. P < 0.05 was considered statistically significant.

## Results

### The expression of miR-124 in breast cancer tissues and adjacent normal tissues analyzed by quantitative real-time PCR

**Figure 1:**
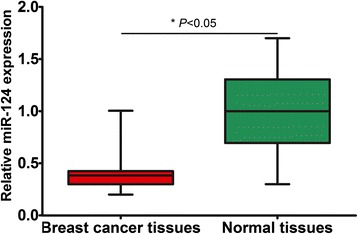
The expression of miR-124 was determined by quantitative real-time PCR in 133 paired human breast cancer and adjacent normal tissues.

Quantitative real-time PCR analysis showed that miR-124 had lower expression in breast cancer specimens than that in matched adjacent normal breast tissues (0.39 ± 0.16 vs. 1.00 ± 0.39; P < 0.05, shown in Figure 1). The median miR-124 expression level of all breast cancer cases was 0.38, which was utilized to divide breast cancer patients into two groups. 66 cases were assigned to the low-expression group, the remaining 67 cases were assigned to the high-expression group.

### The relationship between miR-124 expression level and clinicopathological characteristics

The relationship between miR-124 expression level and clinicopathological characteristics was shown in Table 1. Low miR-124 expression level was significantly associated with advanced TNM stage (P = 0.011), lymph node metastasis (P = 0.012), and poorer pathological differentiation (P = 0.023). However, there was no significant association between miR-124 expression and other clinicopathological factors, including age (P = 0.19), and tumor size (P = 0.13), ER status(P = 0.31), PR status(P = 0.15), and HER2 status(P = 0.08).

### Impact of miR-124 expression on prognosis of breast cancer

**Figure 2:**
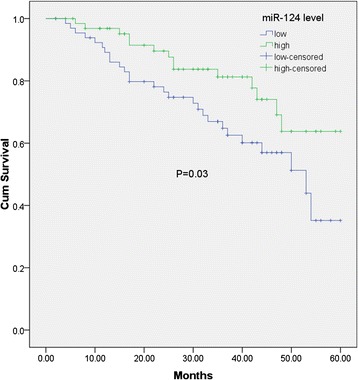
Relationship between miR-124 expression and survival time in breast cancer.

Kaplan–Meier method and log-rank test were used to evaluate the differences of overall survival between low-expression group and high-expression group. A significant difference was found that breast cancer patients with low miR-124 expression level had distinctly shorter overall survival than patients with high miR-124 expression level (63.8% vs. 35.2%, P = 0.03, shown in Figure 2). Univariate and multivariate analyses were utilized to evaluate whether the miR-124 expression level and various clinicopathological features were independent prognostic parameters of breast cancer patient outcomes. The results of analysis are shown in Table 2. A multivariate analysis of the prognosis factors with a Cox proportional hazards model confirmed that low miR-124 expression was a significant independent predictor of poor survival in breast cancer (HR = 3.16, 95% CI: 1.79-9.13, P = 0.017).

**Table 2:** **Univariate and multivariate analyses of different prognostic parameters on breast cancer survival**

	**Univariate analyses**			**Multivariate analyses**		
**Variables**	**HR**	**95% CI**	**P**	**HR**	**95% CI**	**P**
Age	1.45	0.88-4.17	0.32	1.22	0.78-3.44	0.52
Tumor size	1.67	0.78-5.18	0.11	1.39	0.81-4.22	0.18
Lymph node metastasis	3.88	1.87-11.27	0.018	3.36	1.46-10.11	0.029
TNM stage	4.01	1.83-12.18	0.012	3.48	1.59-9.83	0.019
Pathological differentiation	3.32	1.65-9.82	0.005	2.81	1.56-8.87	0.006
ER status	0.67	0.42-3.25	0.31	0.72	0.18-2.03	0.35
PR status	1.52	0.67-3.18	0.27	1.27	0.39-2.88	0.41
HER2 status	1.66	0.25-2.98	0.25	1.91	0.27-3.75	0.22
miR-124 level	2.28	1.28-7.11	0.023	3.16	1.79-9.13	0.017

*P <0.05 was considered significant.

## Discussion

Breast cancer is the most common malignancy in women and is a leading cause of cancer-related deaths among women worldwide. Despite tremendous efforts in developing multimodal treatments, the clinical outcome of breast cancer patients remains unfavorable. The survival time varies considerably between patients. Heterogeneity in both cytology and gene expression makes it difficult to coordinate effective therapeutic strategies which work for every patient. Thus, it is of great significance to realize the underlining molecular mechanisms and to identify powerful prognostic indicator for breast cancer.

### The tumor-related miRNAs function as tumor suppressors or oncogenes and modulate

many aspects of carcinogenesis, including cell proliferation, cell-cycle control, metastasis, as well as angiogenesis [18-20]. Recently, the correlations of dysregulated miRNAs with human breast cancer are increasingly reported, and these data indicate that specific miRNA expression patterns are associated with the biological and clinical properties of breast cancer. However, there have been a limited number of studies on the potential of miRNAs used for prognostic biomarkers and therapeutic molecular targets in breast cancer [21,22].

Previous research confirmed that miR-124 was epigenetically silenced in various types of cancers [12-16]. The expression level and mechanism of miR-124 have also been investigated in breast cancer. Han et al found that miR-124 played a critical role in inhibiting the invasive and metastatic potential of breast cancer cells, probably by directly targeting the CD151 genes. Their findings highlighted an important role of miR-124 in the regulation of invasion and metastasis of breast cancer cells and suggested a potential application for miR-124 in breast cancer treatment [12]. In the study by Li et al, luciferase reporter assay and western blot were used to verify E26 transformation specific-1 (Ets-1) as a potential major target gene of miR-124, and the results showed that miR-124 could bind to putative binding sites within the Ets-1 mRNA 3′ untranslated region (UTR) to reduce its expression. Based on these findings, they proposed that miR-124 and Ets-1 might serve as a therapeutic agent in breast cancer [23]. In another study, Li et al showed that miR-124 was downregulated in breast cancer, and miR-124 might be a tumor suppressor in breast cancer via the regulation of FLOT1 [17]. In the present study, we aimed to investigated the clinical significance of miR-124 in breast cancer. Quantitative real-time PCR analysis showed that miR-124 had lower expression in breast cancer specimens than that in matched adjacent normal breast tissues. Low miR-124 expression level was significantly associated with advanced TNM stage, lymph node metastasis, and poorer pathological differentiation. Kaplan–Meier method and log-rank test were used to evaluate the differences of overall survival between low-expression group and high-expression group. A significant difference was found that breast cancer patients with low miR-124 expression level had distinctly shorter overall survival than patients with high miR-124 expression level. Furthermore, multivariate analysis of the prognosis factors with a Cox proportional hazards model confirmed that low miR-124 expression was a significant independent predictor of poor survival in breast cancer.

## Conclusions

In conclusion, these findings proved that the decreased expression of miR-124 was associated with tumor progression and poor prognosis in patients with breast cancer, suggesting miR-124 might be a novel and valuable signature for predicting the clinical outcome of patients with breast cancer.
